# Common Deregulation of Seven Biological Processes by MicroRNAs in Gastrointestinal Cancers

**DOI:** 10.1038/s41598-018-21573-w

**Published:** 2018-02-19

**Authors:** Lin Zhang, Yuchen Zhang, Sunny H. Wong, Priscilla T. Y. Law, Shan Zhao, Jun Yu, Matthew T. V. Chan, William K. K. Wu

**Affiliations:** 1 Department of Anaesthesia and Intensive Care, The Chinese University of Hong Kong, Hong Kong, China; 2 Institute of Digestive Diseases and State Key Laboratory of Digestive Diseases, LKS Institute of Health Sciences and Department of Medicine and Therapeutics, The Chinese University of Hong Kong, Hong Kong, China; 3 Department of Microbiology, The Chinese University of Hong Kong, Hong Kong, China

## Abstract

MicroRNAs are frequently dysregulated in human neoplasms, including gastrointestinal cancers. Nevertheless, the global influence of microRNA dysregulation on cellular signaling is still unknown. Here we sought to elucidate cellular signaling dysregulation by microRNAs in gastrointestinal cancers at the systems biology level followed by experimental validation. Signature dysregulated microRNAs in gastric, colorectal and liver cancers were defined based on our previous studies. Targets of signature dysregulated miRNAs were predicted using multiple computer algorithms followed by gene enrichment analysis to identify biological processes perturbed by dysregulated microRNAs. Effects of microRNAs on endocytosis were measured by epidermal growth factor (EGF) internalization assay. Our analysis revealed that, aside from well-established cancer-related signaling pathways, several novel pathways, including axon guidance, neurotrophin/nerve growth factor signaling, and endocytosis, were found to be involved in the pathogenesis of gastrointestinal cancers. The regulation of EGF receptor (EGFR) endocytosis by two predicted miRNAs, namely miR-17 and miR-145, was confirmed experimentally. Functionally, miR-145, which blocked EGFR endocytosis, prolonged EGFR membrane signaling and altered responsiveness of colon cancer cells to EGFR-targeting drugs. In conclusion, our analysis depicts a comprehensive picture of cellular signaling dysregulation, including endocytosis, by microRNAs in gastrointestinal cancers.

## Introduction

MicroRNAs (miRNAs) are small non-coding RNAs of 19–23 nucleotides that negatively regulate gene expression through binding to the 3′UTRs of target mRNAs, thereby repressing the translation of target mRNAs into proteins or inducing the degradation of target mRNAs. It has been demonstrated that overexpression of a single miRNA can downregulate over 100 mRNAs, not including targets whose inhibition solely depends on translational repression^1^. Such broad-reaching effect of miRNA on gene expression has been proposed to help define tissue-specific gene expression in humans^1^. In this connection, experimental evidence further supports the notion that a miRNA may target multiple components of the same signaling pathway to exert its action. For instances, the oncogenic miR-17-92 cluster simultaneously targets transforming growth factor β receptor II (TGFBR2), mothers against decapentaplegic 2 (SMAD2) and SMAD4 to regulate TGF-β signaling in neuroblastoma^2^. miR-8 also concomitantly downregulates wntless, CG32767 and TCF to modulate Wnt/β-catenin signaling in *Drosophila*^3^. In this connection, identification of miRNA-regulated pathways by integrative analysis of transcriptomic data is an area of active investigation. Jacobsen and colleagues systematically analyzed the expression of miRNAs and mRNAs across 11 cancer types and identified recurrent miRNA-target relationships and important miRNA-regulated cancer pathways^4^. Ding and colleagues also identified five tumor invasion- and metastasis-related pathways and mitogen-activated protein kinase (MAPK) signaling associated with hepatic cancer stem cells^5^.

The expression of miRNA is dysregulated in most, if not all, types of human neoplasms, including cancers of the digestive tract. The three most common digestive tract cancers, namely, gastric, colorectal, and liver cancers, collectively account for about 23.5 and 27.0% of total incidence and mortality of non-cutaneous malignancies worldwide, respectively^6^. A remarkable number of miRNAs show differential expression in these cancers. In this connection, we previously reported the miRNA expression signatures of gastric, colorectal and liver cancers^7–9^. These dysregulated miRNAs promote oncogenic phenotypes, such as unchecked cell proliferation, evasion of apoptosis, and enhanced metastatic potential. The global influence of miRNA dysregulation on the intracellular signaling network at the systems biology level in these cancers, however, has not been described.

**Figure 1:**
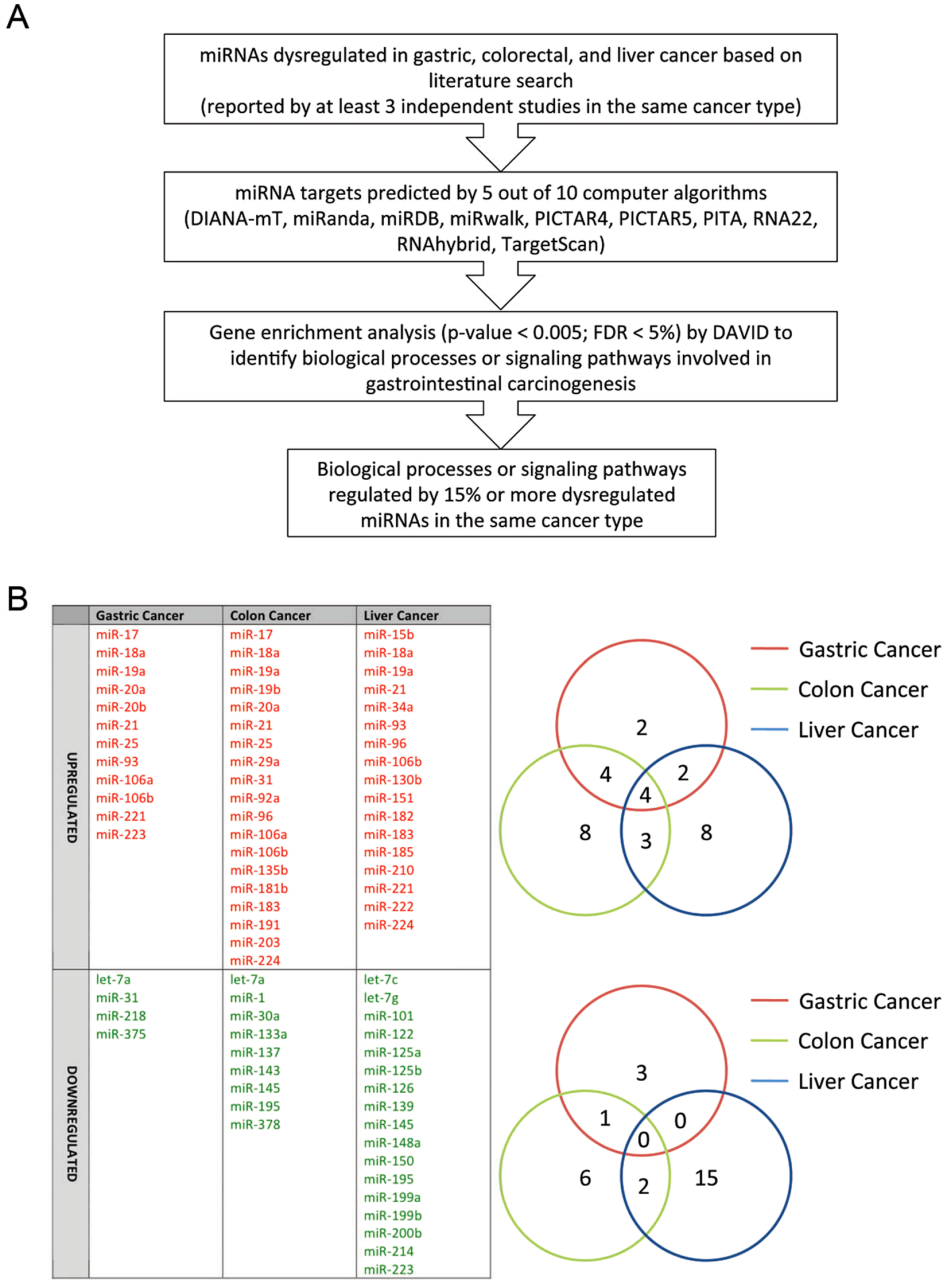
Bioinformatic approach for depicting global signaling deregulation by miRNAs in gastrointestinal cancers. (**A**) Workflow for predicting signaling pathways or biological processes deregulated by signature dysregulated miRNAs in gastric, colorectal and liver cancer. (**B**) Dysregulated miRNAs in gastric, colon and liver cancers. The listed miRNAs are shown to be dysregulated by at least three independent studies.

In this study, we systematically depicted signaling dysregulation by miRNA in human digestive cancers. We first established the tissue miRNA expression signatures in gastric, colorectal, and liver cancers based on our previous reports and then predicted the mRNA targets of these dysregulated miRNA in respective cancer using multiple computational algorithms, followed by gene ontology analysis to determine if the predicted miRNA targets are non-randomly enriched in particular biological processes or signaling pathways (Fig. 1A). As a proof-of-concept, this bioinformatic workflow rediscovered several well-established oncogenic pathways that may be perturbed by dysregulated miRNAs. Surprisingly, our analysis also indicated the involvement of some previously undiscerned or emerging signaling pathways in the pathogenesis of gastrointestinal cancers. The regulation of one of these processes, namely endocytosis, by miRNAs was confirmed experimentally.

## Methods

### Defining miRNA expression signature and miRNA target prediction

We have previously reported the tissue miRNA expression signatures of gastric, colorectal and liver cancers based on literature search^7–9^. Only miRNAs shown to be dysregulated in the same cancer type by at least three independent studies (including microarray studies) were included in the present analysis. To define potential targets of dysregulated miRNAs, ten publicly available computational algorithms, including DIANA-microT, miRanda, miRDB, miRwalk, PICTAR (4-way and 5-way), PITA, RNA22, RNAhybrid, and TargetScan, were used. Candidate genes for subsequent gene ontology analysis were defined as those commonly predicted by at least 5 algorithms.

### Gene ontology analysis

Predicted miRNA target genes were analyzed by The Database for Annotation, Visualization and Integrated Discovery (DAVID) v6.7 via the web-based application program interface. The following annotation categories were included in the HTML links: BBID (a relational database of archived images from research articles that describe regulatory pathways of higher eukaryotes), BIOCARTA (a database of gene interactions within pathways for human cellular processes accessible via Cancer Genome Anatomy Project website), EC_NUMBER (a numerical classification scheme for enzymes, based on the chemical reactions they catalyze), KEGG_PATHWAY (a collection of manually drawn pathway maps representing our knowledge on the molecular interaction and reaction networks)^10,11^, PANTHER_PATHWAY (a database of over 177, primarily signaling, pathways, each with subfamilies and protein sequences mapped to individual pathway components), and REACTOME_PATHWAY (a free, open-source, curated and peer reviewed pathway database). Signaling pathways or biological processes fulfilling the following criteria were considered biologically and statistically significant: (a) *p*-values of Fisher’s exact test <0.005; (b) False discovery rates (FDR) <5% and (c) Being regulated by 15% or more dysregulated miRNAs in the same cancer type.

### Cell culture and cell viability assay

The human colon cancer cell line SW1116 was purchased from the American Type Culture Collection (Manassas, VA). Cells were maintained in RPMI 1640, supplemented with 10% fetal bovine serum, 100 U/mL penicillin and 100 μg/mL streptomycin (all purchased from Life Technologies, Rockville, MD) at 37 °C in a humidified atmosphere of 5% CO_2_ and 95% air. Cell viability was determined by MTT [3-(4,5-dimethylthiazol-2-yl)-2,5-diphenyltetrazolium bromide] assay. Briefly, cells were seeded at a density of 5 × 10^4^ per well on 96-well plate. After treatment, MTT solution (5 mg/mL) was dissolved in culture medium in a ratio of 1:10 and was added to each well for a 4-hour incubation. Dimethyl sulfoxide (DMSO) was added to solubilize MTT tetrazolium crystals. Optical density was determined at 570 nm using a Benchmark Plus microplate reader (Bio-Rad Laboratories, Hercules, USA) and measurement at 630 nm was used for normalization.

### MicroRNA transfection

MicroRNA precursors and microRNA precursor negative control were acquired from Ambion (Life technologies). Twenty-five nanomolar per ml miRNA duplex were mediated into cells by transient transfection using Lipofectamine 2000 (Invitrogen) for 6 hours following the manufacturer’s protocol.

### Quantification of miRNAs with real-Time PCR

Total RNA was isolated using TRIzol reagent (Life Technologies). Reverse transcription and real-time PCR measurement of miRNAs were performed using the MicroRNA Reverse Transcription Kit and TaqMan MicroRNA Assays (Applied Biosystems), respectively. U6 was used as an endogenous control. Total RNA was extracted from three independent experiments, and the real-time PCRs for each miRNA were performed in triplicate.

### EGFR endocytosis assay

Colon cancer cells were transiently transfected with pre-miR-17, pre-miR145 or miRNA precursor negative control for 48 h. Transfected cells were trypsinized and allowed to grow on coverslips in 6-well plates. Following 0.1% serum starvation for 12 h, cells were treated with 2 μg/ml Alexa Fluor EGF (Molecular Probes) for 45 min at 4 °C to recruit ligand-bound receptor at the cell surface, and then warmed to 37 °C for 10 min or 30 min to enable endocytosis. Cells were fixed in 4% paraformaldehyde for 10 min at designated time points, and then washed with phosphate-buffered saline. DAPI staining was used to visualize the nuclei. Fluorescence signal was obtained using confocal microscope.

### Western blots

Cells were harvested in radioimmunoprecipitation buffer containing proteinase and phosphatase inhibitors (Roche, Switzerland). Protein assay kit (Bio-Rad) was used for protein quantitation. Equal amounts of protein were resolved by SDS–PAGE, and transferred to Hybond C nitrocellulose membranes (Amersham Corporation, Arlington Heights, IL, USA). The membranes will be probed with primary antibodies (Abcam for ARF6 and CHMP2B; Cell Signaling Technology for other proteins) overnight at 4 °C and incubated for 1 h with secondary peroxidase-conjugated antibodies. Chemiluminescent signals will then be developed with Lumiglo reagent (Cell Signaling Technology, Netherlands) and detected and quantified by the ChemiDoc XRS gel documentation system (Bio-Rad).

### Statistical analysis

Statistical analysis was performed with Student’s t-test or an analysis of variance (ANOVA) followed by the Tukey’s t-test where appropriate. *P* values less than 0.05 were considered statistically significant.

## Results

### Common miRNA dysregulations in human digestive cancers and their association with cancer pathways

**Figure 2:**
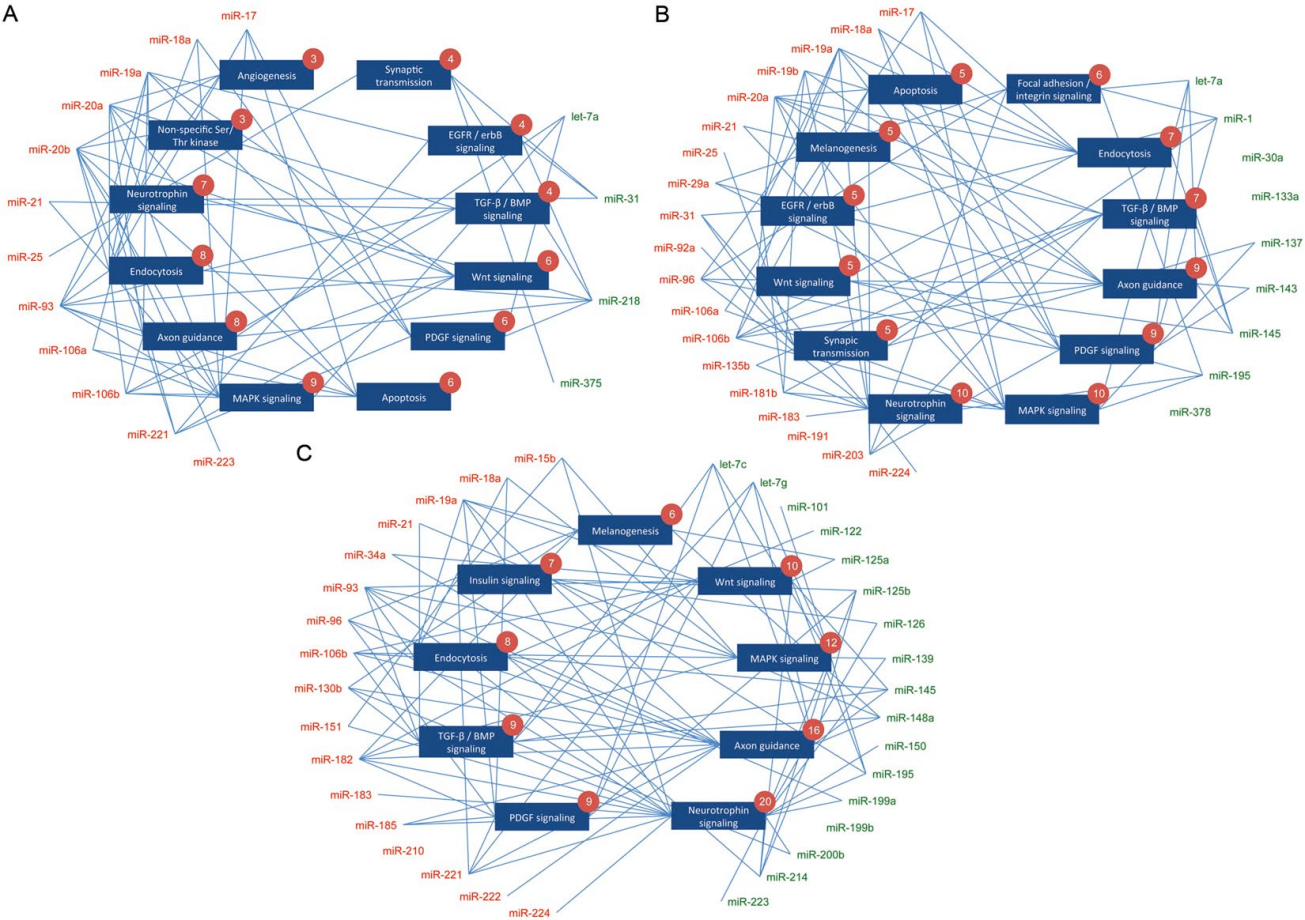
Signaling pathways or biological processes deregulated by ≥15% dysregulated microRNA in gastric (**A**), colorectal (**B**), and liver cancers (**C**). Numbers in the circles indicate the numbers of predicted miRNAs regulating particular pathways.

miRNAs shown to be dysregulated in gastric, colorectal or liver cancer by 3 or more independent studies were shown in Fig. 1B. A total of 16 (12 upregulated; 4 downregulated), 28 (19 upregulated; 9 downregulated) and 34 (17 upregulated; 17 downregulated) miRNAs were found to be dysregulated in gastric, colorectal and liver cancer tissues, respectively. miR-18a, miR-19a, miR-21 and miR-106b were commonly upregulated in all three types of gastrointestinal cancers. miR-145 and miR-195 were commonly downregulated in colorectal and liver cancers whereas let-7a is commonly downregulated in gastric and colorectal cancers. Out of a total of 58 dysregulated miRNAs in gastric (Fig. 2A), colorectal (Fig. 2B), and liver cancers (Fig. 2C), predicted targets of 34 dysregulated miRNAs (58.6%) were found to be associated with “pathways in cancer” or non-specifically with at least one type of malignant diseases. Several established oncogenic signaling pathways, including Wnt/β-catenin signaling, MAPK cascade, transforming growth factor (TGF)-β/bone morphogenetic protein (BMP) signaling, and platelet-derived growth factor (PDGF) signaling, were universally perturbed by dysregulated miRNAs in all three types of cancer. Moreover, we identified miRNA-mediated deregulation of several other cancer-related biological processes, including angiogenesis, apoptosis, epidermal growth factor receptor (EGFR)/ErbB signaling, and insulin signaling in gastric, colorectal or liver cancers.

### Deregulation of established oncogenic signaling by miRNAs in digestive cancers

Both Wnt/β-catenin and Ras-Raf-MAPK cascades are oncogenic in the digestive tract. The Wnt signaling operates by stabilizing the transcription factor subunit β-catenin through repressing the function of β-catenin degradation complex (APC, AXIN and GSK3β) while the Ras-Raf-MAPK cascade can be activated by RAS or RAF mutations or by different signaling molecules such as growth factors, cytokines, G-protein-coupled receptors and integrins^12^. Our bioinformatic analysis estimated that the Wnt/β-catenin signaling was deregulated by 12 out of 58 (20.7%) dysregulated miRNAs in digestive cancers (Table 1). We also found that the Ras-Raf-MAPK cascade might be perturbed by 20 out of 58 (34.5%) dysregulated miRNAs (Table 1).

**Table 1:** Seven signaling pathways or biological processes predicted to be commonly deregulated by miRNAs in gastric, colorectal and liver cancers.

Biological Process	Frequency	Predicted microRNAs
Wnt/β-catenin signaling	12 out of 58 (20.7%)	miR-19a, miR-20a, miR-20b, miR-34a, miR-93, miR-106b, miR-125a, miR-145, miR-182, miR-195, miR-214, miR-221
Ras-Raf-MAPK cascade	20 out of 58 (34.5%)	let-7a/c/g, miR-15b, miR-17, miR-18a, miR-20a/b, miR-21, miR-93, miR-101, miR-106a/b, miR-137, miR-139, miR-181b, miR-182, miR-195, miR-214, miR-224
TGF-β/BMP signaling	13 out of 58 (22.4%)	let-7c/g, miR-20a, miR-21, miR-93, miR-106b, miR-130b, miR-135b, miR-145, miR-148a, miR-181b, miR-200b, miR-203
PDGF signaling	16 out of 58 (27.6%)	let-7a/c/g, miR-1, miR-17, miR-18a, miR-19a, miR-29a, miR-96, miR-125b, miR-143, miR-182, miR-185, miR-203, miR-218, miR-221
Axon-guidance	23 out of 58 (39.7%)	let-7a/c/g, miR-19a/b, miR-20a/b, miR-29a, miR-34a, miR-93, miR-96, miR-106b, miR-125b, miR-130b, miR-143, miR-145, miR-148a, miR-182, miR-185, miR-214, miR-218, miR-221, miR-222
Neurotrophin/NGF signaling	26 out of 58 (44.8%)	miR-15b, miR-19a/b, miR-31, miR-93, miR-96, miR-125b, miR-126, miR-130b, miR-135b, miR-139, miR-150, miR-151, miR-181b, miR-182, miR-183, miR-185, miR-195, miR-199a, miR-200b, miR-203, miR-214, miR-218, miR-221, miR-223, miR-224
Endocytosis	13 out of 58 (22.4%)	miR-17, miR-18a, miR-19a/b, miR-20a/b, miR-93, miR-106b, miR-122, miR-145, miR-199a, miR-214, miR-218

TGF-β/BMP signaling functions as a tumor-suppressing pathway in early stages of gastrointestinal cancers but promotes tumor progression in the late stage^12^. Through our proposed bioinformatic workflow, it was demonstrated that the TGF-β/BMP signaling was expected to be deregulated by 13 out of 58 (22.4%) overexpressed or underexpressed miRNAs (Table 1). Similar to TGF-β/BMP signaling, PDGF signaling is frequently dysregulated in digestive cancers^13–15^. PDGF signaling regulates many cancer-related cellular processes, including cell proliferation, apoptosis, migration, invasion, angiogenesis and metastasis. We here identified that 16 out of 58 (27.6%) dysregulated miRNAs might regulate PDGF signaling (Table 1).

### Unexpected deregulation of axon guidance, neurotrophin/nerve growth factor signaling and endocytosis by miRNAs in digestive cancers

Aside from the abovementioned established oncogenic signaling cascades, our analysis indicated that several unexpected biological processes, including axon guidance, neurotrophin/nerve growth factor (NGF) signaling, and, endocytosis were commonly perturbed by dysregulated miRNAs in gastric, colorectal and liver cancers. Axon guidance is the process by which neurons send out axons to innervate the correct targets. It is now clear that the functions of guidance molecules are not restricted to axon migrations. In this regard, a number of axon-guidance molecules are inactivated in human cancer. Here, we demonstrated that axon-guidance molecules might be deregulated by 23 out of 58 (39.7%) dysregulated miRNAs in digestive cancers (Table 1). Similar to axon-guidance molecules, components of the neurotrophin/NGF signaling are beginning to unveil their importance in human cancer. In this regard, neurotrophin/NGF signaling was predicted to be deregulated by 26 out of 58 (44.8%) dysregulated miRNAs (Table 1). Another biological process highly modulated by dysregulated miRNAs in digestive cancers is endocytosis, which has been implicated in the control of tumor suppression. In this study, we estimated that this putative tumor suppressing process was deregulated by 13 out of 58 (22.4%) dysregulated miRNAs, including miRNAs miR-17 and miR-145, in gastric, colorectal and liver cancers (Table 1).

### MiR-17 and miR-145 alte*r*ed epidermal growth factor receptor endocytosis

**Figure 3:**
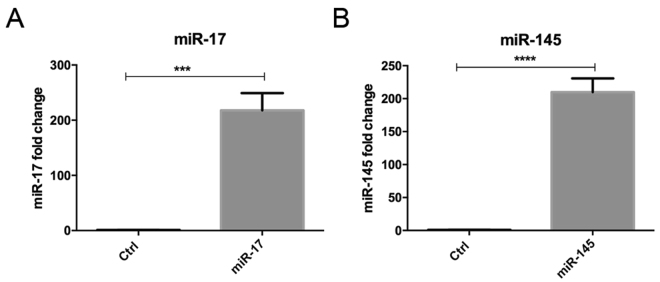
The levels of miR-17 and miR-145 upon transfection with microRNA precursors or negative control were determined by qPCR. ***p < 0.001; ****p < 0.0001 significantly different between groups.

**Figure 4:**
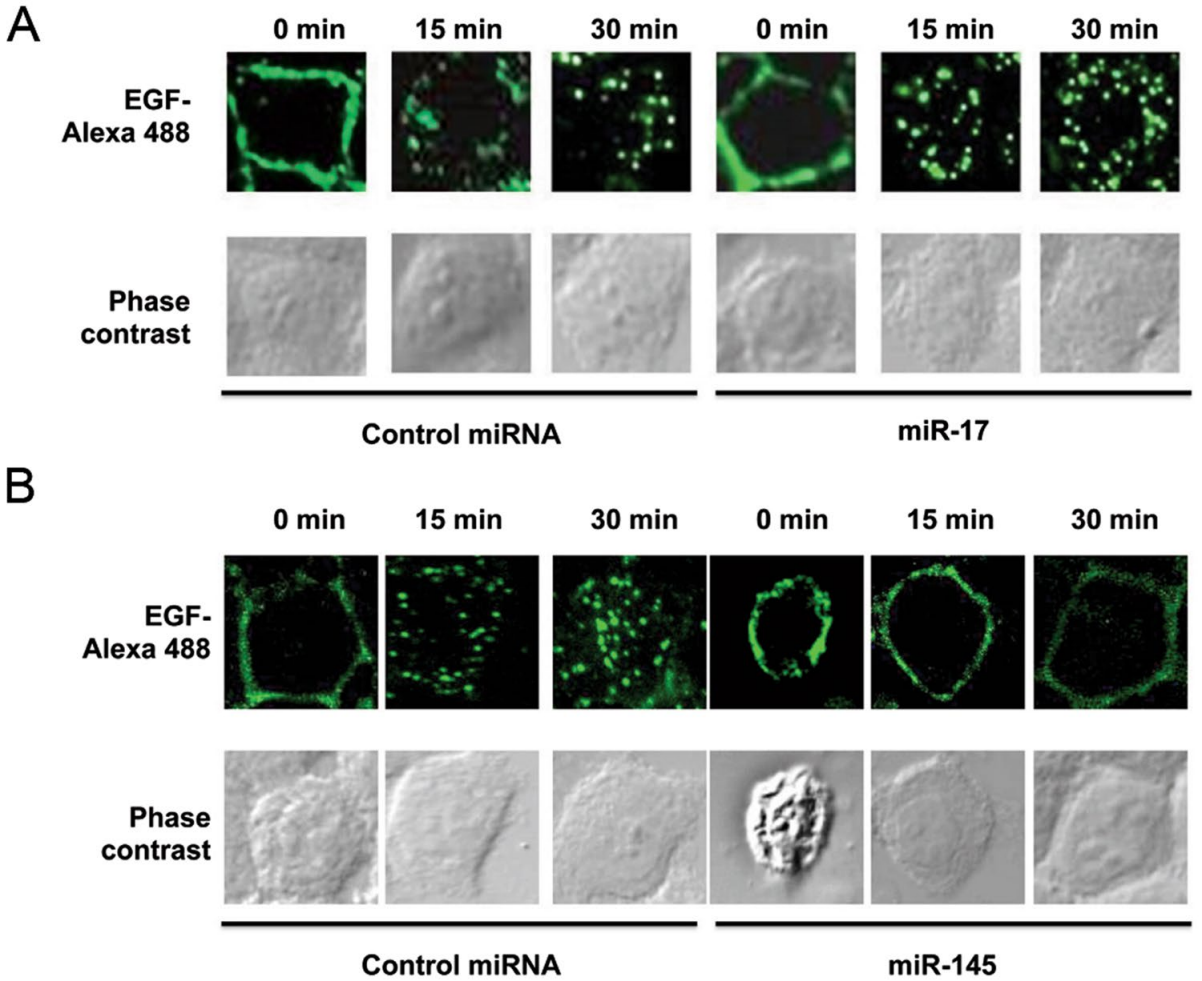
Effects of two predicted miRNAs on EGFR endocytosis. (**A**) MiR-17 accelerated whereas (**B**) miR-145 inhibited internalization of EGFR upon binding of fluorescently labeled EGF. The results are representative of 3 independent experiments.

**Figure 5:**
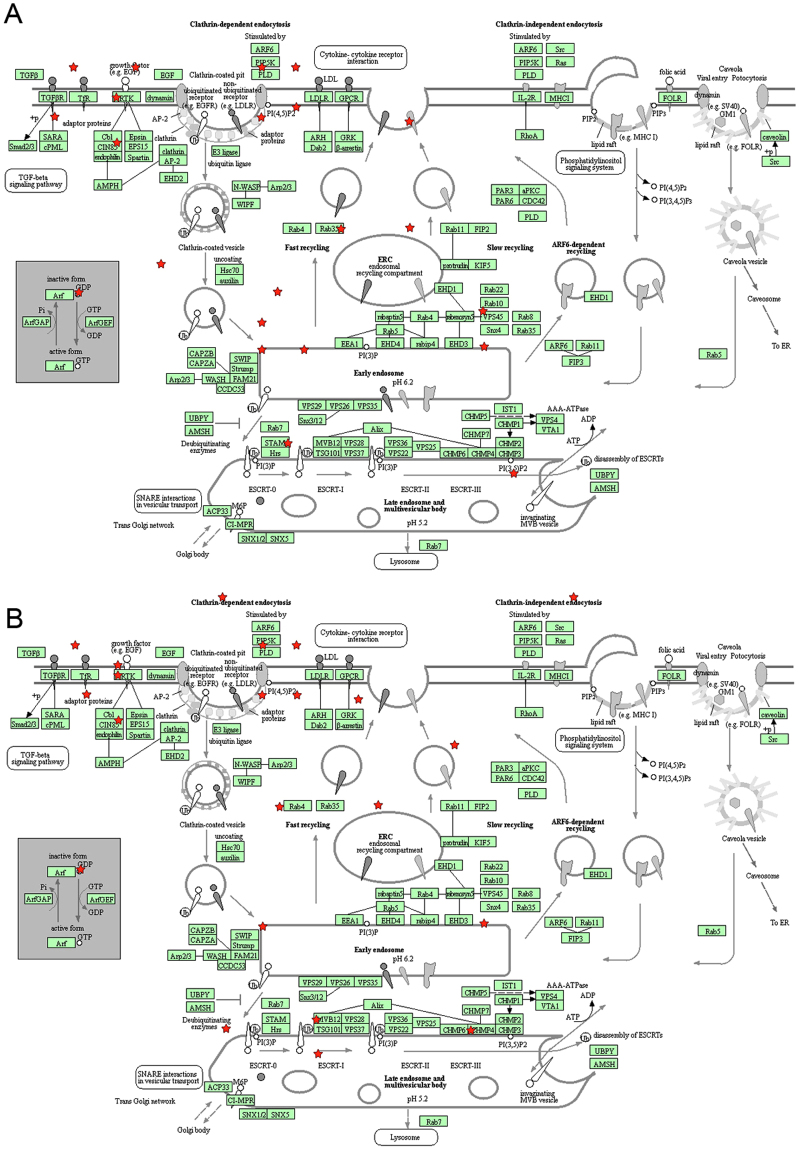
Predicted targets of (**A**) miR-17 and (**B**) miR-145 in the endocytosis pathway. The pathway figures were obtained with permission from KEGG^10,11^.

**Figure 6:**
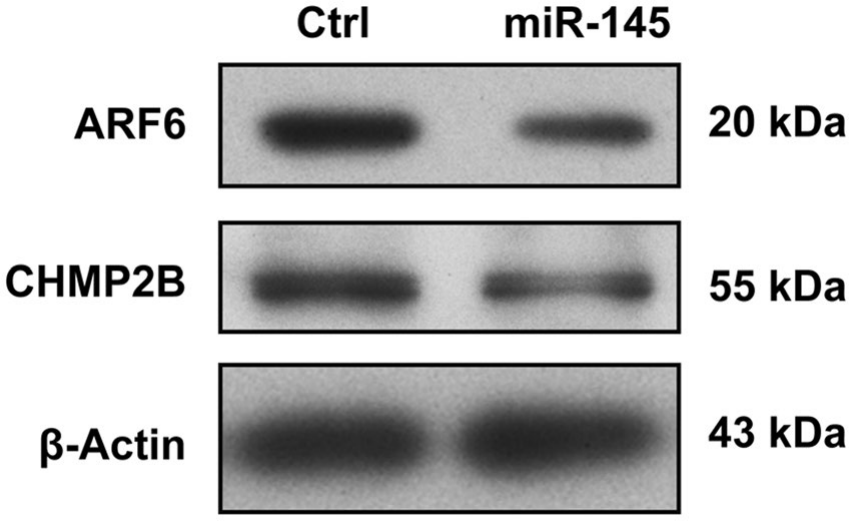
Downregulation of two predicted targets, namely ARF6 and CHMP2B, involved in the endocytic pathway by miR-145 was confirmed by Western blots. The results are representative of 3 independent experiments.

Among these unexpected signaling pathways, the regulation of endocytosis by miRNA in relation to tumorigenesis is the least understood. Since deregulation of epidermal growth factor receptor (EGFR) signaling commonly occurs in colorectal cancer and contributes to its progression, we decided to study how two selected miRNAs, namely miR-17 and miR-145, would affect EGFR endocytosis. Both miR-17 and miR-145 have been identified as two of the most common differentially expressed miRNAs across digestive cancers in our meta-analysis of small RNA sequencing data from The Cancer Genome Atlas^16^. SW1116, a *KRAS*-wild-type colon cancer cell line, was transfected with control miRNA, miR-17 or miR-145. The increased levels of miR-17 and miR-145 after transfection with miRNA precursors were confirmed by RT-qPCR (Fig. 3). Fluorescently labeled-EGF internalization assay was then used to assess EGFR endocytosis. We observed that miR-17 promoted whereas miR-145 inhibited the endocytosis of EGFR upon EGF binding to plasma membrane in SW1116 (Fig. 4). The predicted targets of miR-17 and miR-145 involved in the endocytosis pathway were shown in Fig. 5A,B, respectively. In this regard, the downregulation of two predicted miR-145 targets, namely ARF6 and CHMP2B, involved in the endocytic pathway upon transfection with miR-145 was validated by Western blots (Fig. 6).

### miR-145 prolonged EGFR membrane signaling

**Figure 7:**
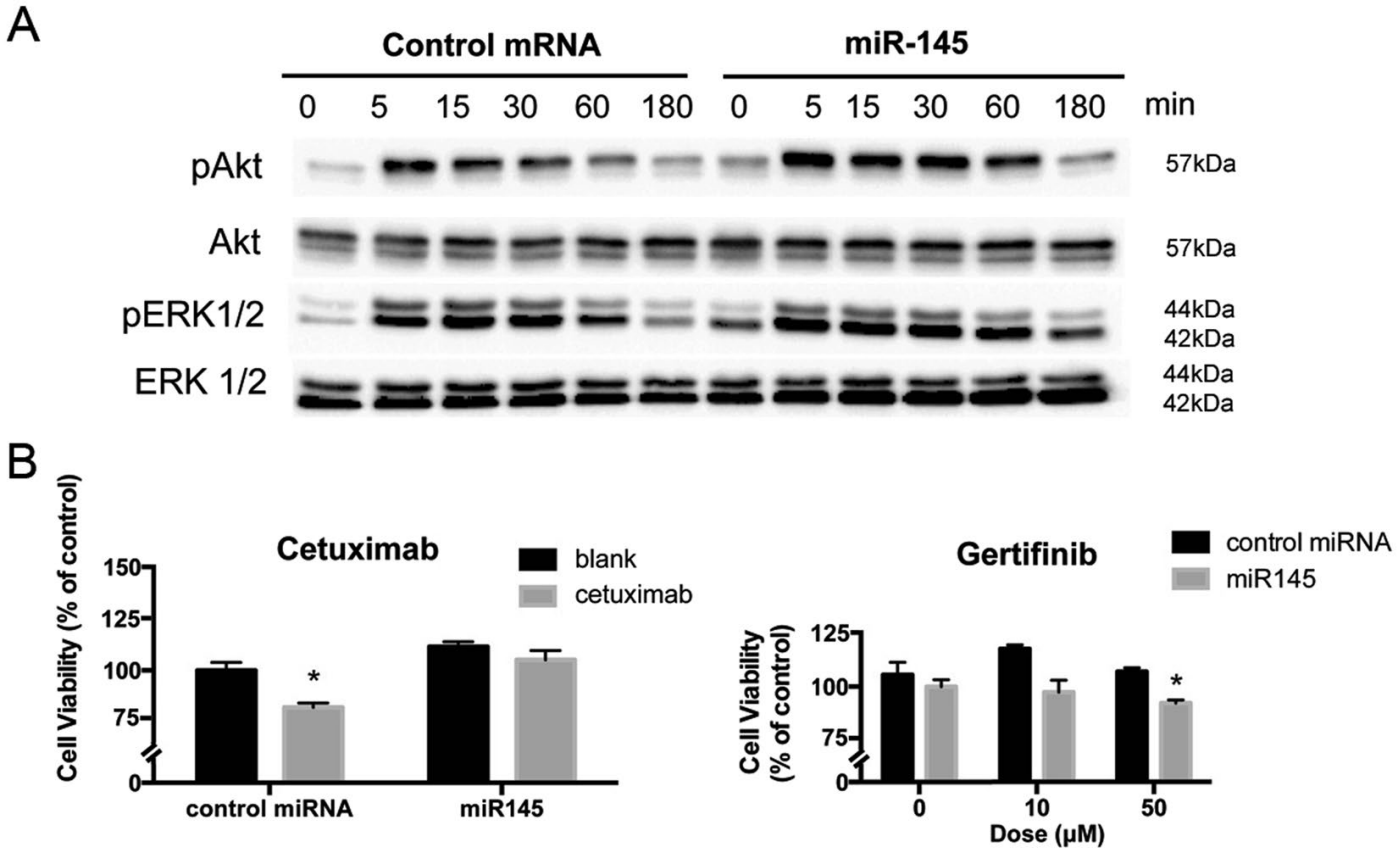
Prolongation of EGFR membrane signaling and alteration of sensitivity to EGFR-targeting drugs by miR-145. (**A**) MiR-145 transfected SW1116 cells showed prolonged increased levels of phosphorylated Akt and ERK1/2 upon stimulation with EGF (50 ng/ml) when compared with control miRNA-transfected cells. (**B**) MiR-145 conferred resistance to cetuximab (100 ng/ml) but increased sensitivity to gertifinib. The results are representative of 3 independent experiments.

To understand the effect of inhibited EGFR endocytosis by miR-145 on EGFR signaling, we examined the activation status of its downstream signaling mediators. Transfected SW1116 cells were treated with EGF (50 ng/ml) and harvested at 0, 30, 60, 90 and 180 min post treatment. As shown in Fig. 7A, in control group, activation of Akt, i.e. phosphorylated Akt (pAkt) level, was the highest 5 min after EGF stimulation and started to fade afterwards, whereas in the miR145-transfected group, p-Akt remained at high level after 60 min. Similarly, pERK1/2 level peaked at 15 min post EGF stimulation and started to drop afterwards in the control group, while in miR145-transfected group, higher levels of p-ERK1/2 were observed at 60 and 180 min post EGF treatments.

### miR-145 altered responsiveness of colon cancer cells to EGFR-targeting drugs

We next assessed the clinical relevance of miR-145-mediated alteration of EGFR endocytosis by measuring cellular response to two classes of EGFR-targeting drugs, namely anti-EGFR antibodies and tyrosine kinase inhibitors (TKIs). The clinical efficacy of the former is known to depend on their ability to trigger receptor downregulation on the cell surface through induction of endocytosis whereas sensitivity to EGFR TKIs increases with heightened basal activities of EGFR downstream mediators, such as p-Akt and pERK1/2^17,18^. Concordant with the literature, miR-145, which blocked EGFR endocytosis and prolonged EGFR membrane signaling, conferred resistance to cetuximab (an anti-EGFR antibody) but increased sensitivity to gertifinib (an EGFR TKI) in SW1116 (Fig. 7B). SW1116 cells were treated with cetuximab or gertifinib for 72 hours before cell viability was measured by MTT assay. Cetuximab induced a significant decrease in cell viability in control miRNA group, whereas no such effect was observed in miR-145 group. In contrast, ectopic miR-145 expression induced a larger reduction in cell viability upon gertifinib treatment compared to control miRNA-transfected cells treated corresponding doses of gertifinib.

## Discussion

miRNA has emerged as a new player in carcinogenesis. In the present study, we predicted that seven biological processes/signaling pathways are commonly perturbed by dysregulated miRNAs in gastric, colorectal, and liver cancers (Table 1), among which four of them, namely, Wnt/β-catenin, Ras-Raf-MAPK, TGF-β/BMP and PDGF signaling are relatively well known for their oncogenic or tumor suppressing properties. Unexpectedly, our bioinformatic analysis also anticipated the involvement of axon guidance, neurotrophin/NGF signaling and endocytosis in the pathogenesis of gastrointestinal cancers. To this end, the regulation of EGFR endocytosis by predicted miRNAs was experimentally verified. Here, we demonstrated that these three biological processes are major targets of dysregulated miRNAs in gastrointestinal cancers. However, there are still several limitations that cannot be overcome in the present study. First, the list of deregulated miRNAs might have been influenced by cohort-related factors, such as ethnicity and etiological association. Second, the impact of confounding factors within a single cohort, including age, gender, tumor grade/stage and co-morbidities (e.g. obesity), cannot be assessed. These might have contributed to varying results among miRNA profiling studies in the literature.

The ability of our analysis to “re-discover” several important cancer-related pathways suggested that our approach provides an accurate depiction of signaling dysregulation by miRNAs in gastrointestinal cancers. Our findings also suggested that miRNA dysregulation is a major contributor to the deregulation of these signaling pathways in gastrointestinal cancers. For instances, we estimated that about one-fifth of dysregulated miRNAs contribute to the deregulation of Wnt/β-catenin signaling in gastrointestinal cancers, among which the effects of miR-34a, miR-145 and miR-221 on Wnt/β-catenin signaling have been confirmed experimentally by other investigators^19–21^. Likewise, about one-third of dysregulated miRNAs were estimated to cause the deregulation of Ras-Raf-MAPK cascade. In this regard, the regulation of Ras-Raf-MAPK signaling by let-7a/g, miR-17, miR-18a, miR-20a, miR-21, miR-101, and miR-214 has been validated^22–29^. Moreover, several dysregulated miRNAs predicted by our algorithms to regulate TGF-β/BMP signaling have also been verified experimentally in other cellular contexts. These miRNAs include miR-20a, miR-21, miR-93, miR-106b, miR-130b, miR-145 and miR-200b^30–36^. The direct effect of signature dysregulated miRNAs on PDGF signaling, however, has not yet been demonstrated.

One of the major findings of the present study is that axon guidance, neurotrophin/NGF signaling and endocytosis are involved in the pathogenesis of gastrointestinal cancers and their deregulations are contributed largely by microRNA dysregulation. While the principle functions or roles of these biological processes in carcinogenesis have not been well established, emerging evidence has hinted their involvement in the development of gastrointestinal cancers. For instance, a repertoire of axon-guidance molecules (e.g. netrin-1, UNC5A, NEO1, RGMA) have been shown to be dysregulated in human cancers, including colorectal cancer^37–39^. SLIT-ROBO signaling, a major axon-guidance signaling axis, is also involved in miR-218-mediated regulation of metastasis and invasion in gastric cancer^40^. In this connection, we predicted that miR-218 could regulate axon guidance molecules in gastrointestinal cancers. Intriguingly, miR-218-mediated regulation of SLIT-ROBO signaling has been shown to have crosstalk with vascular endothelial growth factor (VEGF) signaling in zebrafish^41^. miR-29a, another dysregulated miRNA predicted to regulate axon guidance, has also been experimentally confirmed to target neurone navigator 3 (NAV3)^42^. These evidences along with our findings suggest that dysregulated miRNAs may target axon-guidance molecules in cancer to mediate their oncogenic effects.

Similar to axon-guidance molecules, neurotrophin/NGF signaling is implicated in gastrointestinal carcinogenesis. The low-affinity p75 neurotrophin receptor (p75NTR) and tropomyosin-related kinase (Trk) family serve as receptors for neurotrophins. It has been demonstrated that p75NTR- and Trk-mediated neurotrophin/NGF signaling have opposite roles in carcinogenesis. For example, the expression of p75NTR is decreased in gastric cancers compared with the normal gastric mucosa. p75NTR inhibits gastric cancer proliferation by downregulating cyclin A, cyclin D_1_, cyclin E, cyclin-dependent kinase 2, p-Rb, and PCNA and inhibits the invasion and metastasis of gastric cancer through downregulating nuclear factor κ B-dependent expression of urokinase-type plasminogen activator and matrix metalloproteinase-9^43,44^. A similar downregulation and tumor suppressing effect of p75NTR are observed in liver cancer^45^. In contrast, the expression of TrkA, TrkB and TrkC are upregulated in liver cancer and their expression are correlated with DNA methylation^46^. TrkC also binds to BMP receptor II to suppress the tumor suppressing BMP signaling while the Trk antagonist K252a inhibit proliferation in colon cancer cells^47,48^. Our bioinformatic analysis also indicated that microRNA dysregulation leads to deregulation of neurotrophin/NGF signaling in gastrointestinal cancers. In this regard, the direct targeting of brain-derived neurotrophic factor (BDNF) 3′UTR by miR-195, a miRNA predicted to regulate neurotrophin/NGF signaling, has been validated by other investigators by luciferase assay^49^.

Endocytosis, the process by which cells take up macromolecules, has been increasingly implicated in tumor suppression. For instances, endocytosis is required for the deactivation of mitogenic signaling transduced by receptor tyrosine kinases (RTK), failure of which has been shown to cause tumor formation in epithelial organs in *Drosophila*^50^. In colon cancer, the overexpression of endocytic adaptor Huntington interacting protein 1 (HIP1) is associated with poor clinical outcome^51^. Overexpression of HIP1 interferes with clathrin-mediated internalization, thereby increasing the level of RTK at the plasma membrane^52^. Emerging evidence also connects endocytosis to the control of the cell cycle, apoptosis and asymmetric cell division^53^. In this study, we experimentally confirmed two predicted miRNAs possess the ability to regulate EGFR endocytosis. miR-145, which inhibited EGFR endocytosis, also prolonged EGFR membrane signaling and altered responsiveness of colon cancer cells to EGFR-targeting drugs. Consistent with our findings, Zhong and colleagues demonstrated that miR-145 sensitized lung cancer cells to gefitinib^54^, in which direct targeting of EGFR by miR-145 has been demonstrated^55,56^. However, further experiments by overexpressing a labeled endocytic marker to visualize endocytic vacuoles in the EGF internalization assay would be needed to fully confirm the endocytic internalization of EGFR in miR-17- and miR-145-expressing cells.

Our analysis indicated that endocytosis is commonly deranged in gastrointestinal cancers due to miRNA dysregulation. Nevertheless, the involvement of individual predicted miRNA targets in the action of miR-17 and miR-145 on EGFR endocytosis has not been verified experimentally. It is highly possible that the effects were mediated through concomitant downregulation of multiple targets. An interesting finding of our study is the paradoxical effect of miR-145, a well-established tumor-suppressing miRNA, on EGFR endocytosis. While it is known that MAPK signaling cascade and PI3K activation occur mostly from the plasma membrane, endocytic trafficking of EGFR is known to be required for activation of a subset of nuclear signal transducers^57^. The functional consequence of such a disruption of balance between membrane and nuclear EGFR signaling and its relation to the tumor-suppressing action of miR-145, however, warrant further investigation.

Through the use of bioinformatic approach, we predicted that miRNAs deregulate seven biological processes that are of significance to the pathogenesis of gastrointestinal cancers. Importantly, the regulation of some of these biological processes by predicted miRNAs has been confirmed experimentally by us and other investigators. Taken together, our present work depicted a comprehensive picture of signaling dysregulation by miRNAs in three common gastrointestinal cancers. Our bioinformatic workflow may also be utilized to study the global impact of miRNA dysregulation at the systems biology level in other types of cancer.

## Electronic supplementary material

Full-length blots
