# Supplementary material for: Common Deregulation of Seven Biological Processes by MicroRNAs in Gastrointestinal Cancers
Source: Sci Rep. 2018 Feb 19;8:3287. doi: 10.1038/s41598-018-21573-w (PMC5818544; doi:10.1038/s41598-018-21573-w)

Common Deregulation of Seven Biological Processes by MicroRNAs in Gastrointestinal Cancers

Lin Zhang[1], Yuchen Zhang[1], Sunny H. Wong[2], Priscilla T.Y. Law[3], Shan Zhao[1,2], Jun Yu[2], Matthew T.V. Chan[1], William K.K. Wu[1,2]

[1]Department of Anaesthesia and Intensive Care, The Chinese University of Hong Kong, Hong Kong, China.
[2]Institute of Digestive Diseases and State Key Laboratory of Digestive Diseases, LKS Institute of Health Sciences and Department of Medicine and Therapeutics, The Chinese University of Hong Kong, Hong Kong, China.
[3]Department of Microbiology, The Chinese University of Hong Kong, Hong Kong, China.

Supplementary Information

Full-length blots

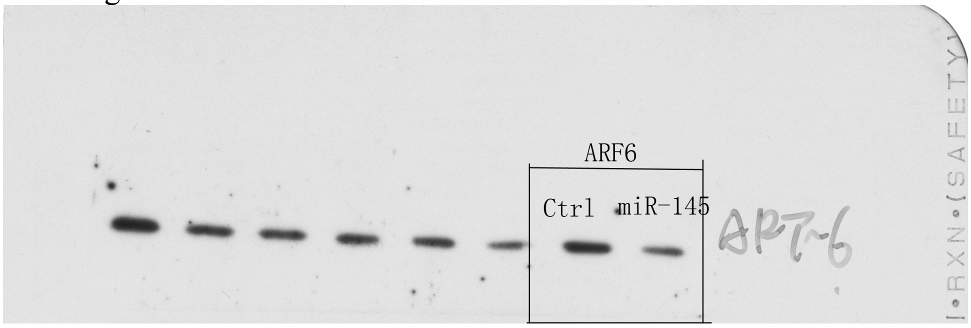

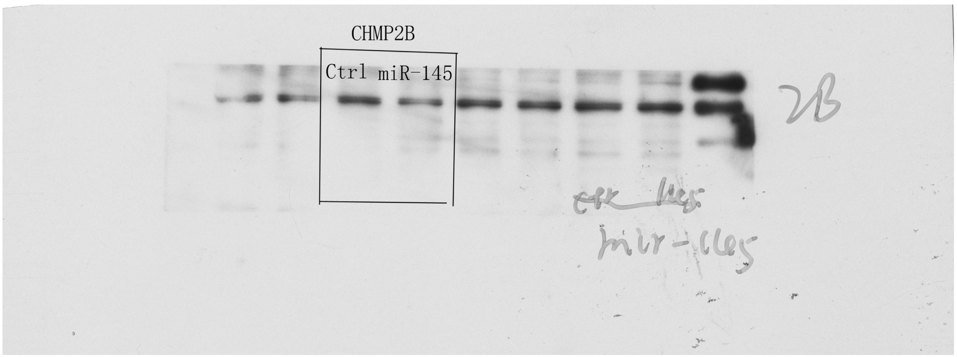

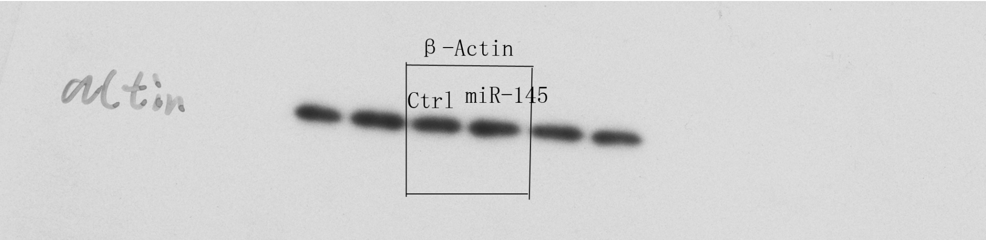

Supplement: Supplementary file 1 — Full-length blots [file 41598_2018_21573_MOESM1_ESM.pdf]
